# Sleep Quality in Head and Neck Cancer

**DOI:** 10.3390/curroncol31110515

**Published:** 2024-11-09

**Authors:** Giancarlo Pecorari, Simone Moglio, Dario Gamba, Marco Briguglio, Ester Cravero, Eugenio Sportoletti Baduel, Giuseppe Riva

**Affiliations:** Division of Otorhinolaryngology, Department of Surgical Sciences, University of Turin, Via Genova 3, 10126 Turin, Italy; giancarlo.pecorari@unito.it (G.P.); simone.moglio@unito.it (S.M.); dario.gamba@unito.it (D.G.); marco.briguglio@unito.it (M.B.); ester.cravero@unito.it (E.C.); eugenio.sportolettibaduel@unito.it (E.S.B.)

**Keywords:** sleep, head and neck cancer, sleep quality, quality of life, insomnia, apnea

## Abstract

Background: Patients with head and neck cancer often experience impaired sleep. Moreover, the treatment may negatively affect sleep quality. The aim of this observational study was to evaluate the sleep quality after treatment for head and neck cancer, and its relationship with quality of life and psychological distress. Methods: A total of 151 patients who underwent treatment for head and neck cancer at our department were included in the study. Quality of life, sleep quality, risk of sleep apnea, sleepiness, pain, and psychological distress were assessed by means of specific questionnaires. Results: The median follow-up was 30 months. Poor sleep quality was observed in 55.6% of the cases. An association between PSQI global sleep quality and EORTC global health status was found. The DT, HADS anxiety, and HADS depression scores were associated to PSQI global score, sleep quality, sleep latency, sleep disturbances, and daytime dysfunction. Conclusions: Sleep disturbances, particularly OSA and insomnia, are frequent in HNC patients, and significantly impact their quality of life and psychological well-being. Given the effect of sleep on overall well-being, addressing sleep disorders should be a priority in the care of HNC patients.

## 1. Introduction

Sleep is essential for cancer patients as it plays a critical role in supporting immune function, emotional health, and the overall quality of life, all of which can impact treatment outcomes. Poor sleep has been linked to increased fatigue, impaired healing, and diminished resilience, making it a key focus in cancer care management [1,2].

Patients with head and neck cancer (HNC) often experience impaired sleep. Sleep quality is closely linked to fatigue, emotional distress, and pain, and it deeply impacts the overall quality of life both before and after treatment [3]. The most common sleep disturbances among this population are insomnia, hypersomnolence, and respiratory sleep disturbances, with the most reported one being obstructive sleep apnea (OSA) [4].

OSA is a common sleep disorder characterized by repetitive episodes of partial or complete upper airway obstruction during sleep. HNC patients are particularly susceptible to OSA due to the anatomical and functional changes caused by both the malignancy and its treatments, such as surgery, radiation therapy, and chemotherapy [5]. These treatments may worsen pre-existing OSA or lead to a new onset of the condition, further contributing to the overall disease burden and the patients’ overall quality of life [6].

Insomnia is a sleep disorder characterized by difficulty falling asleep, staying asleep, or waking up too early and being unable to return to sleep. It can result in poor-quality or insufficient sleep, leading to daytime fatigue, mood disturbances, irritability, and impaired cognitive function. Insomnia is a prevalent issue among patients with HNC; it is often present before and after diagnosis and treatment. Insomnia in these patients is often linked with other complications like pain, fatigue, and emotional distress, significantly impacting their quality of sleep and life [7].

The prevalence of OSA in HNC patients has been reported to range from 57% to 96%, depending on the stage of cancer and the type of treatment received [8]. Moreover, the occurrence of OSA may exacerbate existing comorbidities (e.g., cardiovascular diseases and metabolic syndrome) which are often common in this type of patients [9]. The anatomical distortions caused by tumors or therapeutic interventions, such as radiation-induced fibrosis or surgical alterations to the airway, are key contributors to the development of OSA in this population [8]. Studies have shown that the prevalence of insomnia ranges from 29% before treatment to 45% during treatment and remains at 40% after treatment, indicating a persistent problem throughout the cancer journey [4]. Additionally, psychological factors, such as anxiety and depression, which are common in cancer patients, may also negatively impact sleep quality [10].

Moreover, studies have shown that HNC patients who experienced poor sleep were more likely to suffer from daytime fatigue and reduced cognitive function, further diminishing their ability to manage daily activities [11]. This suggests that managing insomnia and OSA is crucial for improving the overall well-being of head and neck cancer patients during and after treatment. The proper screening, diagnosis, and treatment of OSA, including the use of continuous positive airway pressure (CPAP) therapy, may help alleviate symptoms and improve outcomes for these patients [6]. However, adherence to CPAP therapy remains a significant challenge, with compliance rates being notably low in this patient group [3]. Assessment of distress, anxiety, and depression should be carried on from diagnosis, as well as through and after treatment, and proper psychological management should alleviate the disease burden and lead to a better quality of sleep and thus life. This approach is often overlooked as 82% of patients that report distress, anxiety, and depression do not receive treatment or council to alleviate the problem [12]. Numerous studies have demonstrated that psychological support can substantially improve both the sleep quality and overall well-being in cancer patients. For example, cognitive behavioral therapy has shown significant positive outcomes in managing insomnia and the related symptoms in cancer patients and is recommended to address the dual challenge of psychological distress and sleep disturbance [13,14]. These findings suggest that such interventions could be particularly beneficial in HNC patients, who may face heightened psychological distress.

In conclusion, the interaction between sleep disturbances such as insomnia, obstructive sleep apnea, and HNC poses a significant challenge for clinicians. Both the diseases and their treatment can have profound effects on sleep quality, making it crucial to include sleep and psychological evaluation as a standard component of cancer care. The aim of this observational study was to evaluate the sleep quality after treatment for head and neck cancer. Moreover, it was analyzed in association with quality of life and psychological distress.

## 2. Materials and Methods

A total of 151 patients who underwent treatment for head and neck cancer at our department were included in the study. Exclusion criteria included the following: age < 18 years; follow-up < 12 months; non-epithelial tumors; neurological and/or pre-existing psychiatric disorders that may impact on the ability to fulfill the questionnaires; and active treatment for recurrence or second tumor. Only subjects with a follow-up > 12 months were included in order to analyze patients with stabilized side effects of the treatments. All procedures were in accordance with the ethical standards of the institutional research committee and with the 1964 Helsinki Declaration and its later amendments or comparable ethical standards. Written informed consent was obtained in every case. Institutional Review Board (A.O.U. Città della Salute e della Scienza di Torino—A.O. Ordine Mauriziano—A.S.L. Città di Torino) approval was obtained.

The clinical characteristics, treatment modality, and late radiotherapy toxicity (Radiation Therapy Oncology Group/European Organization for Research and Treatment of Cancer late radiation morbidity scoring system—RTOG/EORTC scale) were recorded. The American Joint Committee of Cancer (AJCC) staging system, 8th edition (TNM—Tumor Node Metastasis), was used for all the tumors. The treatment was based on national and international guidelines.

Quality of life (EORTC QLQ-C30 and EORTC H&N35 questionnaires), sleep quality (Pittsburgh Sleep Quality Index—PSQI), risk of sleep apnea (STOP-BANG), sleepiness (Epworth Sleepiness Scale—ESS), and psychological distress (Distress Thermometer—DT, Hospital Anxiety and Depression Scale—HADS) were assessed. A Visual Analogue Scale (VAS) was used for pain (0 = no pain; 10 = the worst pain). All the questionnaires were administered in person by clinicians.

The EORTC-QLQ C30 questionnaire assessed quality of life through 35 items, evaluating physical, role, emotional, social, and cognitive functioning and somatic symptoms, in addition to the global health score. It was linked with a specific head and neck module (QLQ-H&N35), a 35-item questionnaire that assessed the symptoms encountered specifically by patients with head and neck cancer. Higher symptom scores indicated worse symptoms, while higher scores for global health and functioning scales indicated better quality of life [15,16].

The PSQI questionnaire consisted of 19 items that measure the sleep quality over the previous month. Seven domains (sleep quality, sleep latency, sleep duration, habitual sleep efficiency, sleep disturbances, use of sleeping medications, and daytime dysfunction) were evaluated. Higher scores indicated poorer sleep quality. Taken together, the seven sleep domains were scored as a single factor of global sleep quality (good versus poor): a global score higher than 5 indicated poor sleep quality [17].

The risk of sleep apnea was assessed with the STOP-BANG score, which included 8 dichotomous (yes/no) items (loud snoring, tiredness, observed apnea, systemic hypertension, body mass index—BMI > 35 kg/m^2^, age > 50 years, neck circumference > 40 cm, and male gender). The total score allowed to identify subjects with low, moderate, or high risk of sleep apnea [18].

Sleepiness was evaluated by means of the ESS, which consisted of 8 items representing more or less soporific situations that are scored in a scale of 0–3 (0 = none; 1 = slight; 2 = moderate; 3 = high), resulting in a total score ranging from 0 to 24 points. A higher score suggested a higher propensity to fall asleep (a score above 10 indicated significant daytime sleepiness) [19].

The DT was administered as an easy tool to evaluate the psychological distress by means of a rating scale from 0 (no distress) to 10 (extreme distress), referring to the last week [20]. The HADS questionnaire was administered to assess anxiety and depression and included 14 items, 7 for anxiety and 7 for depression, rated on a 4-point Likert scale (range 0–3). For each subscale, the score was the sum of the respective seven items (range 0–21) [21].

All statistical analyses were carried out using the Statistical Package for Social Sciences, version 26.0. The Kolmogorov–Smirnov test demonstrated a non-Gaussian distribution of variables, so non-parametric tests were used. A descriptive analysis of all data was performed, and they were reported as medians and interquartile range (IQR), or percentages. The Mann–Whitney U test was used for comparison between two independent groups, while the Kruskal–Wallis test was employed for comparison among more than two independent groups. The Chi-squared or Fisher’s exact test was used for categorical variables. Spearman’s test was used to assess the correlation between continuous variables. A *p* value < 0.05 was considered statistically significant.

## 3. Results

The median age was 66 years (IQR 17 years), while the median body mass index (BMI) was 24.86 (IQR 5.09). The median follow-up was 30 months (IQR 49 months). Table 1 highlights the clinical characteristics of the subjects included in the study, whereas Table 2 reports the late radiation toxicity for the patients who underwent radiotherapy.

The median VAS value for pain was 0 (IQR 2). The EORTC QLQ-C30 globally showed a good quality of life (Table 3).

The median ESS score was 1 (IQR 4). The sleep apnea risk evaluated by means of STOP-BANG was low in 74 (49.0%) subjects, intermediate in 52 (34.4%) patients, and high in 25 (16.6%) cases. Table 4 reports the results about sleep quality (PSQI questionnaire). In particular, a poor sleep quality was observed in 84 (55.6%) subjects.

The median DT score was 1 (IQR 4). The HADS questionnaire showed a median anxiety score of 4 (IQR 5) and a median depression score of 4 (IQR 7).

We sought to evaluate the relationship between sleep quality and the other parameters. The PSQI global sleep quality was not statistically related to clinical features, such as sex, age, smoking, alcohol consumption, BMI, tumor site and stage, treatment modality, radiation toxicities, and follow-up (*p* > 0.05). However, women seemed to have a worse sleep quality; a poor sleep quality was observed in 67.4% of women and in 50.5% of men (*p* = 0.054).

Comparing the early and advanced stages, a significant difference was found for PSQI sleep duration and PSQI habitual sleep efficiency (Table 5, *p* < 0.05). No statistically significant difference, in terms of sleep disturbances, was observed among the different treatment modalities (Table 6, *p* > 0.05).

**Figure 1:**
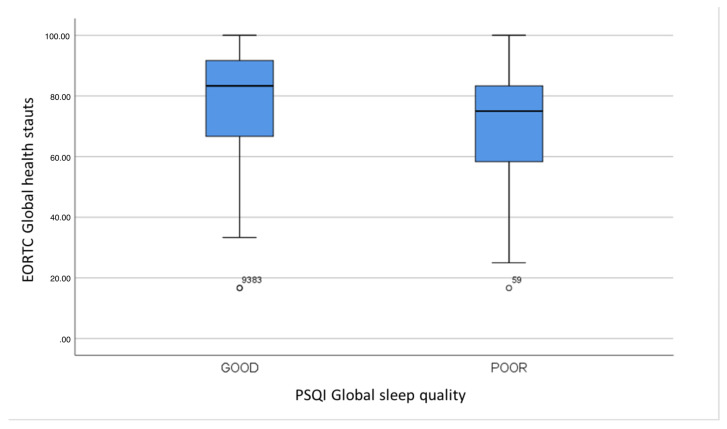
EORTC Global health status in subjects with good and poor sleep quality (*p* = 0.041).

A statistically significant association between PSQI global sleep quality and EORTC global health status was found (Table 7 and Figure 1).

**Figure 2:**
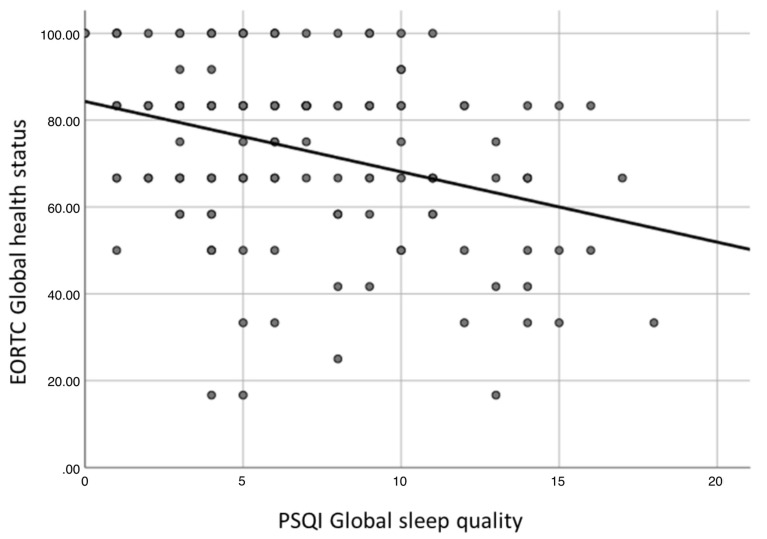
Correlation between PSQI global score and EORTC Global health status (*p* < 0.001).

The correlation tests among PSQI scores and quality of life highlighted the following significant associations (*p* < 0.05):

- PSQI global score with global health status (Figure 2), physical functioning, role functioning, emotional functioning, cognitive functioning, weight gain (inverse correlation), fatigue, pain, dyspnea, insomnia, appetite loss, constipation, diarrhea, social eating, social contact, sexuality, problems opening mouth, dry mouth, sticky saliva, cough, felt ill (direct correlation);
- PSQI sleep quality with global health status, physical functioning, role functioning, emotional functioning, cognitive functioning, social functioning (inverse correlation), fatigue, pain, dyspnea, insomnia, appetite loss, constipation, pain in the mouth, social eating, social contact, sexuality, problems opening mouth, dry mouth, felt ill, weight loss (direct correlation);
- PSQI sleep latency with global health status, physical functioning, role functioning, emotional functioning, cognitive functioning, weight gain (inverse correlation), fatigue, pain, dyspnea, insomnia, appetite loss, constipation, diarrhea, social contact, sexuality, dry mouth (direct correlation);
- PSQI sleep duration with weight gain (inverse correlation), insomnia (direct correlation);
- PSQI habitual sleep efficiency with weight gain (inverse correlation), insomnia, diarrhea, social eating, social contact, sexuality, sticky saliva (direct correlation);
- PSQI sleep disturbances with global health status, physical functioning, emotional functioning, cognitive functioning (inverse correlation), fatigue, pain, dyspnea, insomnia, appetite loss, constipation, diarrhea, financial difficulties, pain in the mouth, swallowing, sexuality, problems opening mouth, dry mouth, sticky saliva, cough, felt ill, weight loss (direct correlation);
- PSQI use of sleeping medications with physical functioning, emotional functioning, (inverse correlation), dyspnea, financial difficulties (direct correlation);
- PSQI daytime dysfunction with global health status, physical functioning, role functioning, emotional functioning, cognitive functioning, social functioning (inverse correlation), fatigue, nausea and vomiting, pain, dyspnea, insomnia, appetite loss, constipation, pain in the mouth, swallowing, social contact, problems opening mouth, dry mouth, sticky saliva, felt ill, weight loss (direct correlation).

**Figure 3:**
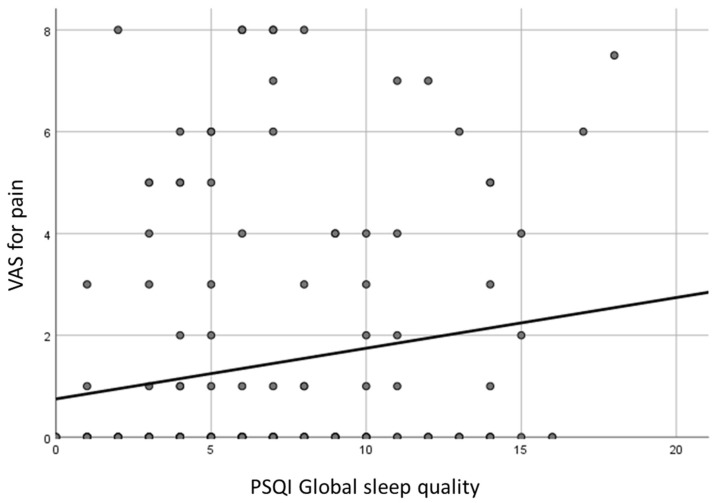
Correlation between PSQI global score and VAS for pain (*p* = 0.036).

The VAS scores for pain were significantly related to the following PSQI scores in a direct relationship: global score, sleep quality, sleep disturbances, daytime dysfunction (*p* < 0.05; Figure 3). 

**Figure 4:**
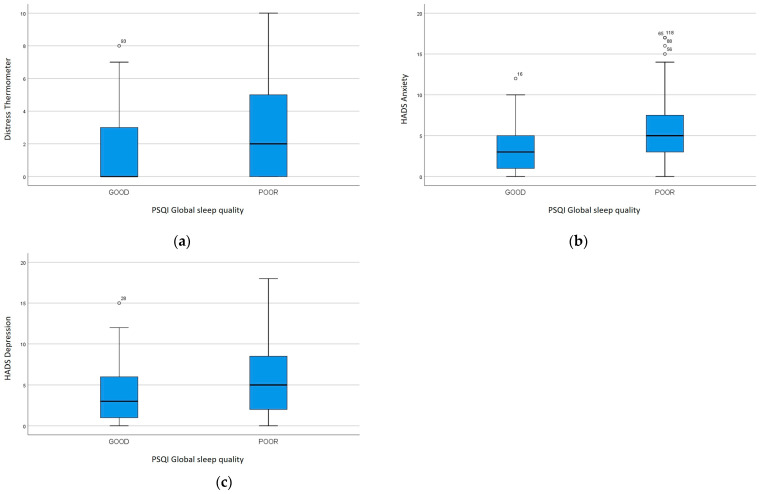
Psychological distress in subjects with good and poor sleep quality: (**a**) DT (*p* < 0.05), (**b**) HADS anxiety (*p* < 0.05), (**c**) HADS depression (*p* < 0.05).

The DT and HADS anxiety scores demonstrated a direct relationship with the PSQI global score, sleep quality, sleep latency, sleep disturbances, use of sleeping medications, daytime dysfunction (*p* < 0.05). The HADS depression score had a direct association with the same PSQI scores (*p* < 0.05), except for the use of sleeping medications. In particular, the DT was 0 (IQR 3) and 2 (IQR 5) in subjects with good or poor sleep quality, respectively (*p* = 0.006). The HADS anxiety score was 3 (IQR 4) and 5 (IQR 5) in patients with good or poor sleep quality, respectively (*p* = 0.001), while the HADS depression score was 3 (IQR 5) and 5 (IQR 7), respectively (*p* = 0.011) (Figure 4).

The ESS score was directly associated with the VAS score for pain and EORTC fatigue and inversely related to the EORTC cognitive and social functioning, PSQI sleep disturbances and daytime dysfunction (*p* < 0.05). It did not have an impact on the DT and HADS scores (*p* > 0.05).

No significant correlation was observed between the risk of sleep apnea and EORTC scores (*p* > 0.05). The PSQI sleep disturbance scores were significantly associated to the STOP-BANG category (*p* = 0.001), being slightly higher in subjects with a high risk of sleep apnea (mean value 1.48), compared to those with intermediate (mean value 1.29) and low risk (mean value 1.05). The STOP-BANG category did not negatively affect the DT and HADS scores (*p* > 0.05).

## 4. Discussion

This study focused on investigating the quality of life, sleep disorders, and their associated factors in patients treated for HNC. Among the most prevalent sleep disturbances in this population were insomnia and OSA, both extensively reported in the literature as the most frequent sleep-related problems affecting individuals with HNC [4]. Similarly, our study confirmed that these two conditions were the primary sleep disorders impacting the patients’ outcomes, contributing significantly to their reduced quality of life.

Our study population of 151 patients consisted primarily of male patients (69.5%), with a median age of 66 years. As expected, a substantial proportion of patients (68.2%) were former smokers. Additionally, 21.2% of patients reported regular alcohol consumption. The demographics and lifestyle factors of our patient cohort were in line with the epidemiology of this type of cancer and other similar studies, further affirming the validity of our sample as representative of the HNC population [8,22,23,24].

When examining the tumor sites in our study, 30.5% of patients were diagnosed with laryngeal and hypopharyngeal tumors, 39.1% had tumors in the oral cavity, 19.9% had oropharyngeal tumors, and the remaining 10.6% were affected by tumors in other sites, such as the salivary glands and nasopharynx. Most patients had an early stage of the disease (50.5%). In terms of treatment modalities, our study revealed that 43.7% of patients underwent surgery as their primary and only form of treatment. In contrast, 17.9% of patients received chemoradiotherapy alone. The remaining 38.4% of patients received a combination of both surgical intervention and chemoradiotherapy.

The overall quality of life in our sample was good with a median score of 83 in the EORTC-QLQ30 Questionnaire. Crucially, the highest score in all the different symptoms affecting quality of life was given to insomnia, signaling that sleep quality was one of the most important factors affecting the perceived quality of life. Pain and fatigue related to treatments are the most probable cause of insomnia, while emotional distress and anxiety related to the disease can also be an aggravating factor in impairing the sleep quality of our patients. This was true in every cohort of the study, but it was especially crucial for patients who underwent radiotherapy as a primary or adjuvant treatment. Indeed, the most common symptom reported in the H&N35 questionnaire was xerostomia, which is strongly related to radiation therapy. Xerostomia is a long-term collateral effect that can impair the quality of life and sleep quality for many years after the end of the treatment. This was in agreement with the literature findings which had analyzed sleep quality, with a particular focus on insomnia and OSA, and the perceived quality of life in HNC patients treated with radiotherapy [22,25]. Artificial saliva can be used to mitigate xerostomia, but this remains by far the most impactful side effect of radiation therapy.

In our study, more than half of the patients (55.6%) reported experiencing poor sleep quality, as indicated by their responses on the PSQI questionnaire. At the same time, the median ESS score was 1, suggesting that, although they experienced no significant daytime hypersomnia, the patients perceived their overall sleep quality to be quite poor. Despite the relatively low ESS score, we observed a direct correlation between the ESS score and the VAS score for pain and EORTC fatigue. Moreover, it was inversely related to cognitive and social functioning. Additionally, a correlation between the ESS and PSQI scores revealed that those with higher ESS scores experienced more disturbances during sleep and reported lower energy levels during the day. Furthermore, we found multiple statistically significant correlations between numerous items of the PSQI and the EORTC-QLQ30 and H&N35 scores, confirming once more the fundamental link between good sleep and good quality of life. The correlation was further confirmed by the direct relationships between the VAS score for pain and the PSQI scores. Everything suggested that even though hypersomnia was not a major concern, it was still closely related to sleep quality in our sample and, in turn, this poorer sleep quality was found to have a negative impact on overall quality of life. These findings were consistent with the previous literature, which had also reported a strong relationship among sleep disturbances, hypersomnia, and the overall quality of life in similar patient populations [11,26]. Our study showed that emotional functioning was the most affected by poor sleep quality, suggesting that clinicians should pay more attention to this aspect of the patients’ life. Although the difference in sleep quality between men and women was not statistically significant (*p* = 0.054), the high proportion of women reporting poor sleep (67.4%) suggested a need for further investigation into gender-specific factors such as hormonal influences and coping differences that may contribute to this pattern. Pain management strategies might alleviate both sleep disturbances and improve the quality of life. Further studies are needed to evaluate how specific types of pain (e.g., neuropathic pain vs. inflammatory pain) respond differently to interventions targeting sleep.

OSA was assessed in our patient population using the STOP-BANG questionnaire. The results revealed that 16.6% of the patients were categorized as being at high risk for OSA, while an additional 34.4% were identified as having a moderate risk. These findings are consistent with the existing literature, which had reported that the prevalence of OSA among HNC patients ranged between 52% and 92%, depending on various factors, such as the stage, the treatment, and the specific location of the tumor [26,27]. This high variability in OSA prevalence is largely attributed to the different anatomical changes that occur in patients undergoing surgery or radiotherapy depending on the specific site of the tumor. These treatments often result in alterations to the airway, such as tissue scarring or structural shifts, which can lead to significant airway obstruction. As a result, these physical changes can exacerbate pre-existing OSA or even induce the condition in patients who were previously unaffected. Our findings highlighted the importance of monitoring for OSA in this patient population, as it could significantly impact not only their sleep quality but also their overall health and recovery [28].

Our study found a statistically significant difference in perceived sleep quality between patients at high risk of OSA and those at lower risk, based on the correlation between STOP-BANG and PSQI scores. Specifically, patients with a higher risk of OSA reported worse sleep quality compared to those in the low-risk group. This finding suggests that OSA, which is more prevalent among HNC individuals [29], has a profound negative impact on sleep quality in this population. The reason for the absence of a significant correlation between the STOP-BANG and EORTC-QLQ30 scores may be the STOP-BANG questionnaire’s inability to adequately capture the severity of sleep apnea in this particular cohort. Indeed, post-treatment anatomical and functional alterations may alter the ability of the STOP-BANG questionnaire to stratify the subjects for OSA risk. Therefore, sleep apnea screening for HNC patients should be based not only on STOP-BANG but also on the type of anatomical and functional alterations due to specific treatments (e.g., partial laryngectomy).

A direct correlation between the psychological scores (DT and HADS) and the PSQI global score was also observed. Poor sleep was strictly connected to anxiety, depression, and emotional distress in our cohort. These psychological factors are known to exacerbate sleep disturbances, as both depression and anxiety are closely linked to insomnia, further compounding the problem of sleep disruption in these patients [30]. Anxiety seemed more associated with medication use compared to depression. The reason may be the association between anxiety and sleep-onset insomnia [31]. This finding stresses the importance of good psychological support for HNC patients as a means not only to improve mental health but also sleep quality. Indeed, disturbed sleep quality can lead to an even worse quality of life. ESS correlated with pain, fatigue, cognitive function, and social functioning. However, the correlation between ESS and psychological distress was not statistically significant. This might imply that daytime sleepiness in these subjects was more physically, rather than emotionally, driven. The low median ESS despite poor sleep quality suggested that, while daytime sleepiness was not a major complaint, sleep disturbances at night were significant. This warrants further exploration to evaluate how patients could be adjusting to chronic poor sleep, or if there were other compensatory mechanisms at play.

The strength of this study was the comprehensive analysis of sleep quality in relationship to the quality of life and psychological distress. The main limitation was the absence of an objective diagnosis of OSA. Indeed, we evaluated the sleep apnea risk only by means of the STOP-BANG questionnaire. Further studies should include objective tools, such as polysomnography. Moreover, longitudinal studies may be useful to assess how sleep quality evolves over time after treatment, especially in relation to ongoing symptoms like xerostomia or psychological distress.

## 5. Conclusions

Sleep disturbances, particularly OSA and insomnia, are frequent in HNC patients, and significantly impact their quality of life and psychological well-being. Our findings are consistent with the existing literature, which highlights the complex relationship among sleep quality, cancer-related symptoms, and treatment-related toxicities. Given the effect of sleep on overall well-being, addressing sleep disorders should be a priority in the care of HNC patients. This study underscores the importance of addressing sleep disturbances in cancer survivors, with a focus on managing the persistent symptoms such as xerostomia, psychological distress, and pain that significantly impact sleep quality and overall well-being. Integrating routine screenings for sleep disturbances, alongside tailored interventions like pain management, psychological support, and treatments for xerostomia, could be effectively implemented in clinical practice. The implementation of screening protocols for sleep disorders, the integration of multidisciplinary teams and expanding intervention strategies, such as behavioral therapies, pharmacological approaches, and sleep-focused rehabilitation, could provide a comprehensive framework to support better sleep quality and quality of life in cancer survivors. Future research should continue to explore the underlying mechanisms of sleep disturbances in this population and to develop targeted interventions to improve sleep quality and, ultimately, patient outcomes.

## Figures and Tables

**Table 1:** Clinical characteristics of the whole sample (*n* = 151).

Characteristics	*n* (%)
Sex	
Male	105 (69.5)
Female	46 (30.5)
Smoking	
Never	35 (23.2)
Former	103 (68.2)
Active	13 (8.6)
Alcohol assumption (>2 drinks for men, >1 drink for women)	
Never	113 (74.8)
Former	6 (4.0)
Active	32 (21.2)
Tumor site	
Oral cavity	59 (39.1)
Oropharynx	30 (19.9)
Larynx/hypopharynx	46 (30.5)
Others	16 (10.6)
Tumor stage	
0	2 (1.3)
I	51 (33.8)
II	27 (17.9)
III	27 (17.9)
IV	31 (20.5)
Not available	13 (8.6)
Treatment modality	
Surgery	66 (43.7)
Surgery and adjuvant RT or CT-RT	58 (38.4)
Exclusive RT or CT-RT	27 (17.9)

CT-RT, Chemoradiotherapy; RT, Radiotherapy.

**Table 2:** Late radiation toxicity in patients who underwent radiotherapy (*n* = 85).

RTOG/EORTC Late Radiation Morbidity Scoring System	*n* (%)
Skin	
0 None	43 (50.6)
1 Slight atrophy, pigmentation change, some hair loss	39 (45.9)
2 Patchy atrophy, moderate teleangiectasia, total hair loss	3 (3.5)
3 Marked atrophy, gross teleangiectasia	0 (0.0)
4 Ulceration	0 (0.0)
5 Death directly related to late effects of radiation	0 (0.0)
Subcutaneous tissue	
0 None	35 (41.1)
1 Slight fibrosis and loss of subcutaneous fat	40 (47.1)
2 Moderate fibrosis but asymptomatic, slight field contracture <10% linear reduction	10 (11.8)
3 Severe fibrosis and loss of subcutaneous, field contracture <10% linear measurements	0 (0.0)
4 Necrosis	0 (0.0)
5 Death directly related to late effects of radiation	0 (0.0)
Mucous membrane	
0 None	31 (36.4)
1 Slight atrophy and dryness	42 (49.4)
2 Moderate atrophy and teleangiectasia, little mucous	10 (11.8)
3 Marked atrophy with complete dryness, severe teleangiectasia	1 (1.2)
4 Ulcerations	1 (1.2)
5 Death directly related to late effects of radiation	0 (0.0)
Salivary gland	
0 None	30 (35.3)
1 Slight dryness of mouth, good response to stimulation	41 (48.2)
2 Moderate dryness of mouth, poor response on stimulation	10 (11.8)
3 Complete dryness of mouth, no response on stimulation	4 (4.7)
4 Fibrosis	0 (0.0)
5 Death directly related to late effects of radiation	0 (0.0)
Larynx	
0 None	52 (61.2)
1 Hoarseness, slight arytenoid edema	20 (23.5)
2 Moderate arytenoid edema, chondritis	0 (0.0)
3 Severe edema, severe chondritis	0 (0.0)
4 Necrosis	0 (0.0)
5 Death directly related to late effects of radiation	0 (0.0)
Not applicable (Total laryngectomy)	13 (15.3)

RTOG/EORTC, Radiation Therapy Oncology Group/European Organization for Research and Treatment of Cancer late radiation morbidity scoring system.

**Table 3:** Results from EORTC QLQ-C30 and EORTC H&N35 questionnaires in the whole sample (*n* = 151).

Scores	Median (IQR)
EORTC QLQ-C30	
Global health status	83.33 (16.67)
Physical functioning	93.33 (20.00)
Role functioning	100.00 (33.33)
Emotional functioning	83.33 (25.00)
Cognitive functioning	100.00 (16.67)
Social functioning	100.00 (33.33)
Fatigue	11.11 (33.33)
Nausea and vomiting	0.00 (0.00)
Pain	0.00 (16.67)
Dyspnea	0.00 (0.00)
Insomnia	0.00 (33.33)
Appetite loss	0.00 (0.00)
Constipation	0.00 (33.33)
Diarrhea	0.00 (0.00)
Financial difficulties	0.00 (0.00)
EORTC H&N35	
Pain in the mouth	0.00 (8.33)
Swallowing	0.00 (16.67)
Senses	0.00 (33.33)
Speech	11.11 (33.33)
Social eating	0.00 (8.33)
Social contact	0.00 (6.67)
Sexuality	0.00 (33.33)
Problems with teeth	0.00 (33.33)
Problems opening mouth	0.00 (33.33)
Dry mouth	33.33 (33.33)
Sticky saliva	0.00 (33.33)
Cough	0.00 (33.33)
Felt ill	0.00 (0.00)
Painkillers	0.00 (0.00)
Nutritional supplements	0.00 (0.00)
Feeding tube	0.00 (0.00)
Weight loss	0.00 (0.00)
Weight gain	0.00 (0.00)

IQR, Interquartile Range.

**Table 4:** Results from PSQI questionnaire in the whole sample (*n* = 151).

PSQI Scores	Median (IQR)
Global score	6 (6)
Sleep quality	1 (1)
Sleep latency	1 (2)
Sleep duration	1 (3)
Habitual sleep efficiency	1 (3)
Sleep disturbances	1 (0)
Use of sleeping medications	0 (0)
Daytime dysfunction	1 (1)

IQR, Interquartile Range.

**Table 5:** Comparison of results from PSQI in patients with early/advanced stage and different treatment modalities (*p* values from Mann–Whitney U test).

PSQI Scores (Median and IQR)	Early Stage	Advanced Stage	*p* Values
Global score	7 (6)	5 (5)	0.088
Sleep quality	1 (1)	1 (1)	0.648
Sleep latency	1 (1)	1 (2)	0.549
Sleep duration	1.50 (3)	0 (2)	0.040 *
Habitual sleep efficiency	1 (3)	0 (3)	0.013 *
Sleep disturbances	1 (0)	1 (0)	0.276
Use of sleeping medications	0 (1)	0.50 (1)	0.899
Daytime dysfunction	1 (1)	1 (1)	0.870

IQR, Interquartile Range. * *p* < 0.05.

**Table 6:** Comparison of results from PSQI in patients with different treatment modalities (*p* values from Kruskal–Wallis test).

PSQI Scores (Median and IQR)	Surgery Alone	Surgery and Radiotherapy	Chemoradiotherapy	*p* Values
Global score	6 (5)	6 (6)	5 (6)	0.652
Sleep quality	1 (1)	1 (0)	1 (1)	0.273
Sleep latency	1 (1)	1 (2)	1 (2)	0.888
Sleep duration	1 (3)	1 (3)	0 (2)	0.536
Habitual sleep efficiency	1 (3)	0.50 (3)	0 (3)	0.304
Sleep disturbances	1 (0)	1 (1)	1 (1)	0.531
Use of sleeping medications	0 (0)	0 (0)	0 (0)	0.980
Daytime dysfunction	0 (1)	1 (1)	1 (1)	0.312

IQR, Interquartile Range.

**Table 7:** Comparison of results from EORTC QLQ-C30 and EORTC H&N35 questionnaires in patients with good and poor sleep quality at PSQI (*p* values from Mann–Whitney U test).

Scores (Median and IQR)	Good Sleep Quality	Poor Sleep Quality	*p* Values
EORTC QLQ-C30			
Global health status	83.33 (25.00)	75.00 (25.00)	0.041 *
Physical functioning	93.33 (20.00)	86.67 (26.67)	0.227
Role functioning	100.00 (16.67)	100.00 (33.33)	0.568
Emotional functioning	91.67 (16.67)	83.33 (33.33)	0.008 *
Cognitive functioning	100.00 (16.67)	100.00 (16.67)	0.169
Social functioning	100.00 (33.33)	100.00 (33.33)	0.352
Fatigue	11.11 (22.22)	11.11 (33.33)	0.072
Nausea and vomiting	0.00 (0.00)	0.00 (0.00)	0.266
Pain	0.00 (16.67)	0.00 (33.33)	0.202
Dyspnea	0.00 (0.00)	0.00 (33.33)	0.045 *
Insomnia	0.00 (0.00)	33.33 (33.33)	<0.001 *
Appetite loss	0.00 (0.00)	0.00 (0.00)	0.216
Constipation	0.00 (33.33)	0.00 (33.33)	0.538
Diarrhoea	0.00 (0.00)	0.00 (0.00)	0.113
Financial difficulties	0.00 (0.00)	0.00 (0.00)	0.444
EORTC H&N35			
Pain in the mouth	0.00 (8.33)	0.00 (16.67)	0.392
Swallowing	0.00 (16.67)	0.00 (16.67)	0.957
Senses	0.00 (33.33)	0.00 (16.67)	0.359
Speech	0.00 (22.22)	11.11 (41.66)	0.130
Social eating	0.00 (8.33)	0.00 (16.67)	0.130
Social contact	0.00 (6.67)	0.00 (13.33)	0.043 *
Sexuality	0.00 (16.67)	0.00 (33.33)	0.049 *
Problems with teeth	0.00 (33.33)	0.00 (33.33)	0.942
Problems opening mouth	0.00 (0.00)	0.00 (33.33)	0.215
Dry mouth	33.33 (33.33)	33.33 (33.33)	0.574
Sticky saliva	0.00 (33.33)	33.33 (33.33)	0.294
Cough	0.00 (0.00)	0.00 (33.33)	0.025 *
Felt ill	0.00 (0.00)	0.00 (0.00)	0.322
Painkillers	0.00 (0.00)	0.00 (0.00)	0.992
Nutritional supplements	0.00 (0.00)	0.00 (0.00)	0.718
Feeding tube	0.00 (0.00)	0.00 (0.00)	0.104
Weight loss	0.00 (0.00)	0.00 (0.00)	0.495
Weight gain	0.00 (0.00)	0.00 (0.00)	<0.001 *

IQR, Interquartile Range. * *p* < 0.05.

## Data Availability

The data presented in this study are available on request from the corresponding author.
